# Dental awareness among pregnant and non-pregnant women in Chhattisgarh

**DOI:** 10.6026/973206300213559

**Published:** 2025-10-31

**Authors:** Manisha Kumari, Vineeta Gupta, Shashank Agrawal, Deepesh Kumar Gupta, Vinitha V, Harsha Goyal

**Affiliations:** 1 Department of Periodontics, Government Dental College, Raipur, Chhattisgarh, India; 2 Department of Oral and Maxillofacial Prosthodontics and Implantology, Government Dental College, Raipur, Chhattisgarh, India

**Keywords:** Pregnancy, women's oral health, dental awareness, maternal health, periodontitis

## Abstract

A dental awareness, knowledge and oral hygiene practice among 77 pregnant and 77 non-pregnant women in Chhattisgarh is of interest. A significant lack of awareness was observed, with 100% of non-pregnant and 96.1% of pregnant women not prioritizing routine dental visits and only 3.9% of pregnant women aware of the link between oral health and pregnancy outcomes. Thus, data shows an urgent need to promote oral hygiene and dental care during pregnancy to reduce risks such as preterm birth and low birth weight.

## Background:

The 1998 theme of the World Health Organization, "Safe Motherhood: Pregnancy is Precious-Let's Make It Special," emphasizes the importance of taking extra precautions when pregnant [1]. Both reversible and irreversible alterations are brought about by pregnancy in the oral cavity, which is affected by changes in hormones, especially high levels of estrogen and progesterone [2]. Hormonal changes associated with pregnancy and poor tooth hygiene increase the risk of oral diseases such periodontitis and gingivitis [3]. Several studies have connected periodontal disease to worse pregnancy outcomes, including low birth weight and preterm birth [4, 5-6]. During labor, uterine contractions depend on inflammatory cytokines such as TNF-α, PGE2 and IL-1. Premature increase of these mediators brought on by a periodontal infection, however, may cause early labor and raise the risk of preterm delivery [7, 8- 9]. Offenbacher *et al.* were the first to report that maternal periodontal disease could significantly elevate the risk of PLBW, suggesting a sevenfold increase in likelihood [10]. Similarly, Radnai *et al.* found that periodontal therapy before the 35th gestational week could reduce the risk of adverse pregnancy outcomes [11]. Despite emerging evidence, awareness among healthcare professionals remains low. According to a research by Tarannum *et al.* only 63% of gynaecologists, 56.4% of general dentists and 67.4% of general medical practitioners knew that periodontal disease and PLBW were related [12]. Additionally, research shows that nearly 50% of pregnant women do not seek dental care, even when they believe it is necessary [13]. A South Indian study further reported that despite experiencing dental issues, pregnant women rarely visit a dentist [14]. The reluctance to seek dental care may be influenced by socioeconomic, demographic, psychological and behavioral factors [14]. Therefore, it is of interest to compare the knowledge, attitudes and practices (KAP) related to dental awareness among pregnant and non-pregnant women in Chhattisgarh.

## Materials and Methods:

## Study design and ethical approval:

A cross-sectional survey using questionnaires was carried out in a hospital to gauge dental knowledge in both pregnant and non-pregnant women. The Government Dental College in Raipur's Ethical Committee granted ethical permission for the study (ethical clearance number: ECR/25/GDC/CG/2023). Additionally, permission to conduct the study in the Obstetrics and Gynaecology Outpatient Department (OPD) at Dr. Bhim Rao Ambedkar Memorial Hospital in Raipur was acquired from the Dean of Pt. Jawaharlal Nehru Medical College in Raipur.

## Study population and data collection:

The Department of Obstetrics and Gynaecology at the Dr. Bhim Rao Ambedkar Memorial Hospital in Raipur, Chhattisgarh and the Department of Periodontology at the Government Dental College in Raipur were the sources of the participants. Both pregnant and non-pregnant female OPD patients were included in the study.

## Inclusion criteria:

[1] Pregnant and non-pregnant women visiting the hospital.

[2] Women who voluntarily agreed to participate.

[3] Ability to comprehend and respond to the questionnaire.

## Exclusion criteria:

[1] Women with systemic conditions affecting oral health.

[2] Patients with cognitive or communication impairments.

A pilot study and previously published research served as the foundation for the development of a structured questionnaire [15]. Women who were illiterate or had trouble understanding the questions were given an oral explanation in their native tongue language. Demographic details such as age, educational status and occupation were also recorded.

## Statistical analysis:

Using SPSS for Windows (version 16.0; SPSS Inc., Chicago, IL, USA), data was examined. Categorical variables were summarised using descriptive statistics as n (%). For categorical data comparison, the Pearson Chi-square test was utilised. The significance level for all two-sided statistical tests was set at α = 0.05.

## Results:

Participants' oral hygiene habits, knowledge, attitudes towards oral health, awareness of gum disease, government regulations for maternal oral health care and use of prenatal dental appointments were all evaluated by the questionnaire. Most participants in both groups reported favourable oral hygiene habits. 88.3% of pregnant women (Group A) and 94.9% of non-pregnant women (Group B) used toothpaste for cleaning their teeth as stated in Table 1 (see PDF). To evaluate both groups' knowledge and the connection between oral and systemic health, a chi-square test was used. (Results are considered non-significant if p > 0.05 and statistically significant if p < 0.05). This article provides an overview of the current body of knowledge about the relation between pregnant women's poor dental health and unfavourable pregnancy outcomes as shown in Table 3 (see PDF).

[1] 23.4% of pregnant women in Group A reported a history of abortion.

[2] 1.3% reported a previous preterm birth.

Oral hygiene practices were generally favorable, with a high percentage of women using toothpaste and regularly replacing toothbrushes. Awareness of government maternal health schemes was low, with only 20.8% aware of PMSMA and PMMVY (Table 2 - see PDF). More than half (57.9%) had been approached by healthcare workers regarding maternal oral health care. 4. Only 3.9% of expectant mothers were aware that dental health and pregnancy outcomes are related. Only 15.6% believed that dental check-ups are safe during pregnancy, indicating a significant gap in awareness. Bleeding gums (11.7%) and gum swelling (2.6%) were reported, but only 64.9% sought dental care. A significant proportion (36.4%) believed that pyorrhoea is normal in pregnancy, showing a need for oral health education (Table 4 - see PDF).

## Discussion:

Despite consistent attendance at prenatal appointments, this study found that none of the pregnant participants sought dental care, except for 15.6% of pregnant and 14.1% of non-pregnant females who consulted a dentist. About 41% of women who were not pregnant and 70.1% of pregnant women felt that they did not need to see a dentist. These results are consistent with earlier research showing that 96.1% of pregnant women did not receive regular dental examinations. The study also examined the oral hygiene practices of the sample, including the frequency, method. Most individuals in the study group demonstrated positive oral hygiene practices, with a significant majority engaging in favourable tooth brushing habits. Regarding the frequency of daily tooth brushing among the participants, a majority of 61 % among pregnant females and 52.60% among non-pregnant females (n=115) brushed their teeth twice a day. Furthermore, 49.40% pregnant and 46.20% non-pregnant females among the participants replaced their toothbrush in a month and 19.50% pregnant females replaced their toothbrush when it can no longer be used effectively. A combined 89.60% of pregnant females and 96.20% non-pregnant participants utilized both a brush and toothpaste in their tooth-brushing routine, with 7.80% pregnant and 3.80%non-pregnant participants relying solely on datum. Majority of the participants in both groups had a habit of rinsing after meals except 18.20% pregnant and 6.40% non-pregnant females lacked the same habit. Adverse pregnancy outcomes result from a complex interplay of various factors. These encompass childbearing age, racial background, socioeconomic status, maternal education, tobacco use, inadequate nutrition, the challenge of multiple pregnancies, a history of previous unfavorable obstetric outcomes and genitourinary infections. The increased risk of premature delivery and low birth weight babies has been linked to periodontal disease, a noteworthy new risk factor [16]. However, in the study, both pregnant and non-pregnant women said they understood that poor dental health could affect their unborn child. However, they lacked awareness regarding the specific adverse pregnancy outcomes associated with poor oral health. In 1992, Robinson and Amar evaluated the effects of pregnancy on oral health and found four oral pathological diseases: periodontitis, dental caries and pregnancy granuloma and pregnancy gingivitis.

Machuca *et al.* (1999) showed that 68% of the subjects in a study of 130 pregnant women had gingivitis. Depending on the employment sector, prevalence rates varied from 46% among technical executives to 88% among manual laborers. Cross-sectional studies of pregnant and postpartum women have demonstrated a substantial link between pregnancy and a higher prevalence of gingivitis than the postpartum period, even when plaque assessments were comparable (Silness & Loe, 1963). An analysis of a Sri Lankan rural community of women (Tilakaratne *et al.* 2000) revealed that all of the pregnant women in the study had higher gingivitis, with differing degrees of significance in comparison to matched non-pregnant controls. Most participants consistently attended their scheduled prenatal appointments. However, when queried about dental visits during pregnancy, none of the participants reported receiving advice from their gynaecologist to consult a dentist. Furthermore, they lacked motivation or perceived the necessity of visiting a dentist during pregnancy. Periodontal illnesses are typified by phases of quiescence and exacerbation. Until teeth and oral structures are irreversibly damaged, the diseases are frequently misdiagnosed. Therefore, managing and maintaining periodontal health depend heavily on knowledge and awareness of periodontal disease. This is especially important for expectant mothers, who may be more susceptible to periodontal disease than women who are not expecting [17]. The American Academy of Periodontology (AAP) published a position statement in 2004 that provides guidelines for dental treatment for expectant mothers. The American Academy of Paediatrics promoted preventative dental care for all pregnant or soon-to-be pregnant women, which included prophylaxis, restorative therapy and a periodontal evaluation. Regardless of gestational age, they stressed the need to treat acute infections or abscesses right away and specifically recommended that scaling and root planing be done early in the second trimester. These suggestions were made with the intention of quickly removing any possible sources of infection in order to protect the mother's and the unborn child's health [18]. Dental procedures, including diagnostic measures, periodontal treatments, restorations and extractions, are considered safe and most advisable during the second trimester when organogenesis is concluded [19, 20]. According to this survey, 87% of pregnant women and 11.70% of non-pregnant women were unaware of the connection between poor dental health and overall systemic health. It was documented that physicians did not refer pregnant females for dental checkups when they were getting their regular medical check-up done. The bulk of those surveyed reported generally good oral hygiene practices, brushing their teeth at least once with toothpaste and cleaning them after each meal. Only 20.80% of pregnant women and 30.80% of non-pregnant women are aware of any government programs for maternal care, such as Pradhan Mantri Surakshit Matritva Abhiyan (PMSMA) and Pradhan Mantri Matru Vandana Yojana (PMMVY). There is slight awareness of Chhattisgarh Minimata Mahtari Jatan Yojana 57.10% among pregnant and 60.30% among non-preganant females. These findings are similar to one the study by Sandya Patil, Rachna Thakur et al in 2013 whose findings revealed that gynecologists lacking knowledge (38.8%) and time (27.7%) regarding the implications of poor oral health outcomes were less inclined to recommend comprehensive oral care services for pregnant patients. I nterestingly, 85.7% of gynecologists (p>0.05) reported never examining the oral cavity of patients during routine checkups [21]. Gynecologists' lack of knowledge (38.8%) and time (27.7%) were key barriers to recommending dental care for pregnant patients, as reported by Patil *et al.* (2013). Furthermore, 85.7% of gynaecologists never performed an oral examination as part of a standard examination [21]. While some pregnant women recognized the importance of dental care, many did not associate poor oral health with adverse pregnancy outcomes. Raising awareness is crucial to preventing these complications. While the some of women are aware that they require extra dental care while pregnant, not all of them believe that poor oral health could be the cause of unfavourable pregnancy outcomes. It is important to take steps to raise awareness of this connection in an effort to stop unfavourable pregnancy outcomes from happening.

## Limitations:

Since this is a questionnaire study, it has its own set of constraints. Because of the limited sample size, care should be taken when interpreting the results. The degree of maternal knowledge about dental health and other factors including spacing out children, receiving prenatal care and the mother's nutritional status were not evaluated. The current study, being questionnaire-based in its design, has the limitation that the oral health status of the participants was not clinically assessed or examined through their dental records.

## Conclusion:

General physicians and gynecologists should encourage all pregnant women to schedule dental visits. This is due to potential connections between periodontal disease and overall health as well as between a mother's oral health and that of her foetus. Additionally, pregnant women should heed the advice of oral health professionals regarding proper brushing techniques and the significance of follow-up visits. Awareness about government schemes should also be there so that pregnant women can utilize their benefits.
